# Immunoproteomic Analysis of the Excretory-Secretory Proteins from *Spirometra mansoni* Sparganum

**Published:** 2013

**Authors:** Dan Dan HU, Jing CUI, Li WANG, Li Na LIU, Tong WEI, Zhong Quan WANG

**Affiliations:** Dept. of Parasitology, Medical College, Zhengzhou University, Zhengzhou, P. R. China

**Keywords:** *Spirometra mansoni*, Plerocercoid, (sparganum), Excretory-secretory, Diagnosis

## Abstract

**Background:**

Sparganosis is caused by the invasion of *Spirometra* sparganum into various tissues/organs. Subcutaneous sparganosis can be diagnosed by biopsy, while visceral/cerebral sparganosis is not easy to be diagnosed. The diagnosis depends largely on the detection of specific anti-sparganum antibodies. The specificity of the ELISA could be increased by using *S. mansoni* sparganum excretory–secretory (ES) antigens, but it also had the cross-reactions with sera of patients with cysticercosis or paragonimiasis. The aim of this study was to identify early specific diagnostic antigens in *S. mansoni* sparganum ES proteins.

**Methods:**

The sparganum ES proteins were analyzed by two-dimensional electrophoresis (2-DE) and Western blot probed with early sera from infected mice at 14 days post-infection. The immunoreactive protein spots were characterized by MALDI-TOF/ TOF-MS.

**Results:**

A total of approximately 149 proteins spots were detected with isoelectric point (pI) varying from 3 to 7.5 and molecular weight from 20 to 115 kDa and seven protein spots with molecular weight of 23-31 kDa were recognized by the infection sera. Three of seven spots were successfully identified and characterized as the same *S. mansoni* protein (cysteine protease), and the proteins of other 4 spots were not included in the databases.

**Conclusion:**

The cysteine protease from *S. mansoni* ES proteins recognized by early infection sera might be the early diagnostic antigens for sparganosis.

## Introduction

*Spirometra mansoni* (syn. *Spirometra erinacei* or *S. erinaceieuropaei*) is an intestinal tapeworm of wild and domesticated carnivores; its larvae-plerocercoids (spargana) cause sparganosis. Human is an accidental host. Human infections were mainly acquired by drinking raw water contaminated with cyclops harboring procercoids, ingesting raw flesh of frogs and snakes infected with plerocercoids, or placing frog or snake flesh on open wound for treatment of skin ulcers or eye inflammations (1–3). Sparganosis is sporadically distributed worldwide, but most cases occur in Southeast Asia, Japan, Korea and Thailand; less commonly in the United States and Europe (4, 5). In the People's Republic of China, sparganosis is an important food-borne parasitic zoonosis, with more than 1,000 human cases reported in 27 out of 34 provinces, autonomous regions, or municipal districts during 1927–2009 (6). Recently, the autochthonous cases with sparganosis caused by ingestion of live tadpoles have recently emerged in Henan Province of central China (7). Sparganosis poses a serious threat to human health; the plerocercoids usually lodge in the subcutaneous tissues and muscles, but sometimes invade the abdominal cavity, eye, central nervous system causing blindness, seizures, headache, epilepsy, paralysis, and even death (8, 9).

The clinical diagnosis of sparganosis is rather difficult and often misdiagnosed because the specific signs or symptoms are lacking. A definite diagnosis of subcutaneous sparganosis can be achieved by detection of the larvae in a biopsy specimen from the lesion, but the confirmative diagnosis is very difficult for visceral and cerebral sparganosis since the larva is found only by surgical removal (10). The enzyme-linked immunosorbent assay (ELISA) is rapid and is the most commonly used serological method for the detection of sparganum infection in humans (11). The ELISA using crude antigen of plerocercoids has high sensitivity, but the main disadvantage is the false negative results during the early stage of infection and the cross-reactions with serum samples from patients with other parasitic diseases (cysticercosis, paragonimiasis, clonorchiasis, etc.) (12). Although the specificity of the ELISA was improved when the antigens purified from the crude extract by affinity chromatography or the excretory–secretory (ES) antigens were used, the cross-reactions with sera of patients with cysticercosis or paragonimiasis still occurred (13–15). The results of SDS-PAGE demonstrated that the protein components of the sparganum crude and ES antigens were complicated, and had 19 and 7 bands, respectively (16). The detection of specific IgG4 antibodies against 31 and 36 kDa proteins in sparganum crude antigens appeared to be reliable, but these molecules was cross-reacted with sera of patients with cysticercosis, and showed somewhat blurred, irregular and broad banding patterns (17, 18).

Immunoproteomics, a technique involving two-dimensional electrophoresis (2-DE), followed by immunoblotting, is an approach to identify specific antigenic proteins in high resolution in a wide range of proteins expressed by different organisms (19, 20). The combination of proteomics with Western blotting may support discovery of numerous novel antigens and will help us to screen novel serological diagnostic markers and vaccine candidates. The approach proved to be highly successful in characterizing expressed proteins from other parasitic organisms, such as *Toxoplasma gondi*, *Neospora caninum*, *Eimeria tenella*, *Schistosoma japonicum*, and *Trichinella spiralis* (21–23). In the analysis of *S. mansoni*, previous studies have addressed the differential protein expression in different developmental stages and the new findings may provide insight into *Spirometra* development and biology (24). However, to our knowledge, no ES antigens of *S. mansoni* plerocercoids have been analyzed by immunoproteomics and identified by tandem mass spectrometry.

The purpose of this study was to identify the early diagnostic antigens in *S. mansoni* plerocercoid ES proteins recognized by early infection sera. The ES proteins from plerocercoids were analyzed by two-dimensional electrophoresis (2-DE) and Western blot probed with early sera from the sparganum-infected mice at 14 days post infection (dpi). Then, the immunoreactive protein spots were identified and characterized by Matrix-assisted laser desorption ionization (MALDI)-time-of-flight (TOF)/-TOF-MS analyses in combination with bioinformatics analysis.

## Materials and Methods

### Parasite and experimental animals

Plerocercoids (spargana) of *S. mansoni* were collected from subcutaneous tissue and muscles of the naturally infected wild frogs (*Rana limnocharis* and *Rana nigromaculata*) which were captured from the suburbs of Zhengzhou city of Henan Province, China. The collected worms were washed five times in physiological saline for further inoculating to mice. Forty-day-old specific pathogen-free (SPF) Kunming mice weighing 20 to 25 g were purchased from the Experimental Animal Center of Henan province and bred in plastic micro-isolator cages were used for the study. All procedures of animal experiment of this study were approved by the Life Science Ethics Committee of Zhengzhou University.

### Collection of spargana and infection sera

After their blood being collected, sixty male SPF Kunming mice were inoculated each with two spargana by gastric intubation with physiological saline with the animals under ether anesthesia. From 6 to 28 days post-infection (dpi), 100 µl of tail vein blood was collected on alternate days from each infected animal. All of the infected mice were sacrificed at 28 dpi by deep ether anesthesia, and their carcasses were then skinned. The spargana were recovered for preparation of ES antigens.

### Preparation of sparganum ES antigens

The preparation of ES antigens of spargana was performed as previously described (15). Briefly, after washing thoroughly in sterile normal saline solution and serum-free RPMI-1640 medium supplemented with 100 U penicillin per milliliter and 100 U streptomycin per milliliter, the spargana were incubated in a 75 cm^2^ culture dish with the same medium at concentration of five worms/10 ml for 18 h at 37°C in 5% CO_2_. After incubation, the media that contained the ES products were filtered through a 0.2-µm membrane into a 50-ml conical tube and then centrifuged at 4°C, 15,000×g for 30 min. The supernatant was dialyzed and then lyophilized by a vacuum concentration and freeze-drying (Heto Mxi-Dry-Lyo, Denmark). The ES antigens were diluted to a concentration of 0.71 mg/ml and stored at -20°C before use.

### 2-DE

The ES proteins were precipitated using trichloroacetic acid (TCA) and acetone as previously described method with some modi-fications (25). Briefly, the sample was suspended in 10% TCA in acetone for 3 h at -20°C. After centrifugation at 15 000 g for 15 min at 4 °C, the pellet was resuspended in cold acetone washed for four times. The final pellet was air-dried. The 2-DE was performed as previously described (26). Briefly, the pellet was suspended in rehydration buffer [7M urea, 2M thiourea, 4% CHAPS, 65 mM DTT, 0.2% IPG buffer (pH 3-10) and 0.001% bromophenol blue], containing 700 µg of the protein samples in a total volume of 200 µl and centrifuged at 15 000 g for 10 min at room temperature to remove the insoluble materials. The supernatant was loaded onto 11-cm pH 3-10 immobilized pH gradient (IPG) strips (Bio-Rad, USA) by over-night reswelling and separated by isoelectric focusing (IEF) using a Protean IEF Cell (Bio-Rad, USA). IEF was performed using a Protean IEF Cell at 20 °C as follows: S1: 50 V, 12h; S2:250 V, 30 min;S3: 1 000 V, 30 min; S4: 8 000 V, 4 h; and S5: 8 000V, 40 000 Vh (using a limit of 50 µA/strip). After IEF, the IPG strips were equilibrated sequentially, first in equilibration buffer (6 M urea, 0.375 M Tris-HCl pH 8.8, 2% SDS and 20% glycerol) containing 2% dithiothreitol, then in equilibration buffer containing 2.5% iodoacetamide. The second dimension was performed on 12% SDS-PAGE using a Mini Protean cell (Bio-Rad, USA). Proteins were separated for 30 min at 10 mA and then at 23 mA until the dye front reached the bottom of the gel at 16°C. Three replicates were run for the sample. After 2D gel electrophoresis, proteins were either stained with Coomassie blue R-250 for proteomic analysis or used for immunoblotting as previously described (27). The tests were repeated three times.

### Western blot analysis

Proteins from 2-DE gels were transferred onto polyvinylidene difluoride membrane (PVDF) membranes by semi-dry transfer cell (Bio-Rad, USA) for 1 h at 20 V (27). The membranes were blocked with 5% skimmed milk at 37°C for 1 h. Following three washes with TBST, the membranes were incubated with sera of infected mice (1:100 dilutions) overnight at 4°C. After washing with TBST, blots were then incubated with horseradish peroxidase (HRP)-conjugated goat anti-mouse IgG (Sigma, USA) (1: 6, 000 dilutions) at 37 °C for 1 h. Following three additional washes, the membranes were developed with the enhanced chemiluminescent (ECL) kit (CWBIO, China). Sera collected from mice before infection were used as controls. Immunoblot experiments were conducted in triplicate, with no variation in results observed. Images of immunoblots were captured using ImageScanner (GE healthcare, USA) and aligned with equivalent protein stained 2-DE gels using Image Master 2D Platinum 6.0 (GE healthcare, USA).

### 2-DE gel excision and tryptic digestion

2-DE gel electrophoresis protein spots recognized by early infection sera at 14 dpi were prepared for MALDI-TOF/TOF-MS analysis according to standard protocols (28). Seven immunoreactive spots were excised manually from the Coomassie blue-stained gels. The excised gel pieces carrying the spots were placed in a tube, destained for 20 min in 200 mmol/L NH_4_HCO_3_/30% acetonitrile and then lyophilized. All the lyophilized samples were digested overnight at 37°C× with 12.5 ng/ml trypsin in 25 mmol/L NH_4_HCO_3_. The peptides were extracted three times with 60% ACN/0.1% trifluoroacetic acid (TFA). The extracts were pooled and dried completely by centrifugal lyophilization.

### Protein identification by MALDI-TOF/-TOF-MS

The resulting peptides from the above extraction were analyzed by MALDI-TOF/TOF-MS. The procedure was performed as described previously (29). Briefly, The purified tryptic peptide samples were spotted onto stainless steel sample target plates and mixed (1:1 ratio) with a matrix consisting of a saturated solution of a-cyano-4- hydroxy-trans-cinnamic acid in 50% acetonitrile-1% TFA. Peptide mass spectra were obtained on an Applied Biosystem Sciex 4800 MALDI-TOF/TOF mass spectrometer (Applied Biosystems, USA). Data were acquired using a CalMix5 standard to calibrate the instrument (ABI4700 Calibration Mixture). The MS spectra were recorded in reflector mode in a mass range from 800 to 4000 with a focus mass of 2000. For MS/MS spectra, up to 10 of the most abundant precursor ions per sample were selected as precursors for MS/MS acquisition, excluding the trypsin autolysis peaks and the matrix ion signals. In MS/MS positive ion mode, for one main MS spectrum, 50 subspectra with 50 shots per subspectrum were accumulated using a random search pattern. Collision energy was 2 kV, collision gas was air, and default calibration was set by using the Glu1-Fibrino-peptide B ([M + H] + 1, 570.6696) spotted onto Cal 7 positions of the MALDI target. Combined peptide mass fingerprinting (PMF) and MS/MS queries were performed by using the MASCOT search engine 2.2 (Matrix Science, UK) and submitted to MASCOT Sequence Query server (http://www. matrixscience. com) for identification against nonredundant NCBI database (http://www. ncbi. nlm. nih. gov/BLAST) with the following parameter settings: 100 ppm mass accuracy, trypsin cleavage (one missed cleavage allowed), carbamidomethylation set as fixed modification, oxidation of methionine was allowed as variable modification, and MS/MS fragment tolerance was set to 0.4 Da. The criteria for successfully identified proteins were as follows: ion score confidence index (CI) for peptide mass fingerprint and MS/MS data was ≥95%.

## Results

### 2-DE analysis of sparganum ES antigens

**Fig. 1:**
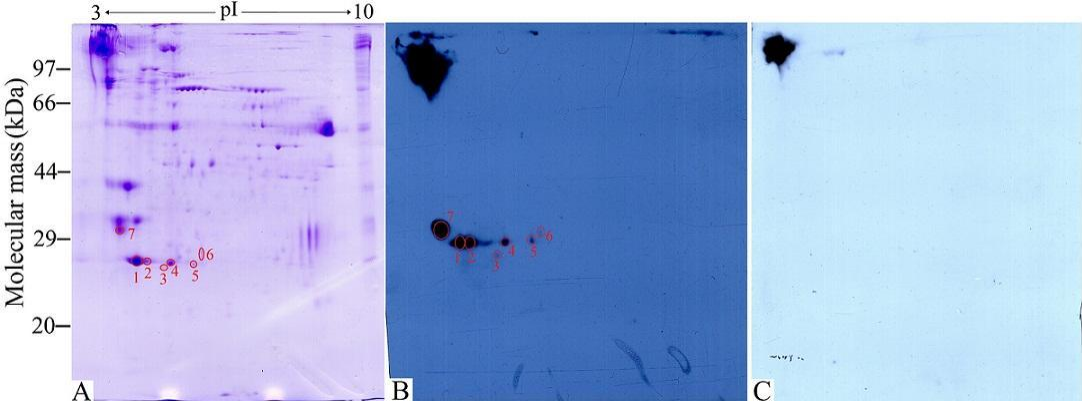
**Two-dimensional electrophoresis (2-DE) and Western blot analysis of** ***Spirometra mansoni*** **sparganum excretory-secretory (ES) proteins**. (A) 2-DE gel of ES proteins separated in the first dimension in the pH range 3-10 and then in the second dimension on a 12% polyacrylamide gel. The gel was stained with Coomassie blue R-250, molecular weight standard is on the left, and pI values are indicated. Protein spots selected for analysis are numbered. (B) 2-DE Western blot of ES proteins probed by mouse infection sera at 14 days post infection (dpi), and the immunoreactive protein spots were detected by the enhanced chemiluminescent (ECL) kit. (C) Western blot map probed by sera from mice before infection.

The ES proteins of *S. mansoni* spargana were separated on a 2-DE gel covering a pH 3-10 nonlinear, and the protein spots were visualized following Coomasie R-250 staining (Fig. 1A). A total of approximately 149 spots were detected on the Coomassie bule stained 2-DE gels, with pI varying from 3 to 7.5 and molecular weight from 20 to 115 kDa. Major protein spots were located in acidic range (pH 3-7) migrating at 24-79 kDa. The 2-DE was repeated three times, and the patterns were highly reproducible.

### Western blot analysis of S. mansoni sparganum ES proteins following 2-DE

Anti-sparganum IgG antibodies in sera from infected mice at 6-28 dpi were assayed by ELISA and Western blot (15). The specific antibodies were firstly detected at 8 dpi and the antibody positive rate was 100% at 14 dpi and persisted until the end of this experiment (28 dpi, data not shown). The infection sera at 14 dpi were used to detect the fractions of sparganum ES proteins. As shown in Fig. 1B, there were 7 spots recognized by the infection sera at 14 dpi. Once photographed, the immunoblot and their homologous Coomassie blue-stained gel were aligned and then matched by Image Master 2D Platinum 6.0 software and artificial recognition, and these 7 positive spots were found to be precisely matched with the homologous gel. The 7 matched positive spots named as spot 1 to 7 with 23-31 kDa were selected to be further analyzed by MS. However, the sera collected from mice before infection did not show detectable immune-reactivity with any of the protein spots (Fig. 1C).

### Identification of immunoreactive proteins by MALDI-TOF/TOF-MS

Out of all 7 matched protein spots recognized by infection sera at 14 dpi, 3 spots (spot 1, 2 and 5) were successfully identified and characterized to correlate with the same *S. mansoni* protein-cysteine proteinase. The three spots had the same molecular mass (24.3 kDa) with different isoelectric point(4.65, 4.79 and 5.54). Additionally, their MOWSE core and coverage were also different. The proteins of other 4 spots were identified as protein of other organism species and not matched with that of *S. mansoni* in the NCBI database. The results of protein identification are shown in Table 1.

**Table 1:** Identification of *Spirometra mansoni* sparganum ES proteins recognized by mouse infection sera at 14 days post infection using MALDI-TOF/TOF-MS

Spot No.	Protein name	Accession No.	Theoretical pI/Mr	Observed pI/Mr	MOWSE score	Coverage (%)	Matched peptides	Species
**1**	cysteine proteinase	gi∣15146346	4.66/20.8	4.65/24.3	187	61	5	*S. mansoni*
**2**	cysteine proteinase	gi∣15146346	4.66/20.8	4.79/24.3	193	19	4	*S. mansoni*
**3**	GH18868	gi∣195036204	6.92/60.14	5.1/23.3	66	40	14	*Drosophila grimshawi*
**4**	Cysteine proteinase rd21a precursor	gi∣149392651	4.81/25.9	5.2/24.2	63	24	3	*Oryza sativa*
**5**	cysteine proteinase	gi∣15146346	4.66/20.8	5.54/24.3	144	15	4	*S. mansoni*
**6**	Chain A, C-Terminal Domain of Transcriptional	gi∣20150171	4.86/7.09	5.58/25.7	72	74	5	*E. coli*
**7**	Suppressor of IKK-epsilon	gi∣213512244	5.03/24.8	4.32/31.4	68	15	5	*Salmo salar*

## Discussions

Although the incidence of sparganosis is relative low, the medical treatment of sparganosis is highly limited, and praziquantel has not obvious effects on sparganosis which requires surgical removal (30–32). Therefore, the diagnosis and differential diagnosis of sparganosis from other causes of the space occupying lesion is important. Plerocercoids can survive for decades in the host tissues and stimulate the host immune system to develop a specific immune response, including a Th2-type response (2). Therefore, anti-sparganum antibodies in sera have been considered useful biomarkers for the diagnosis of sparganosis. For this reason, the early sera of the sparganum-infected mice were used to probe the ES protein fractions of spargana following separation via 2-DE to screen candidates for diagnosis based on proteomics.

In this study, our results also demonstrated a protein pattern of the *S. mansoni* sparganum ES proteins migrating between 20-115 kDa and major protein spots were located in acidic range (pH 3-7) migrating at 24-79 kDa (as shown in Fig. 1A). Seven ES protein spots with 23-31 kDa recognized by sera at 14 dpi were selected to be further identified by mass spectrometry. The results showed that out of 7 protein spots, 3 protein spots with 24.3 kDa were successfully identified by MALDI-TOF/TOF-MS. The 3 protein spots identified represented only the same one protein (cysteine proteinase). The results suggested that the protein which had the same molecular weights with different pI values might be expressed as paralous or allelic forms, or that some of these proteins might be processed by alternative splicing, post-translational modifications and protein processing (29, 33). These modifications could be related to phosphorylation or acetylation of the proteins after translation, and they could be vital for the protein's biological functions, such as parasite survival, immune escape and immunopathogenesis. A previous study has also demonstrated that *T. spiralis* may express more than one isoforms of the protein and that a common precursor protein could undergo variable post-translational processing (34). The cysteine proteinase of *Paragonium westermani* and some low-molecular weight proteins of cysticercus solium were shown to be expressed as multiple isoforms as well as gylcosylated forms (35, 36), and this might be the case for the sparganum ES proteins.

Cysteine protease has been detected in *S. mansoni* (37, 38). It has been shown that the protease of many parasites acts extracellularly to help invade tissues and cells, to uptake nutrient, to hatch or to evade the host immune system. Cysteine protease is the key factor in the parasitic pathogenicity, either by inducing tissue damage and facilitating invasion or by empowering the parasites to salvage metabolites from host proteins (39). The plerocercoids of *S. mansoni* are also known to secrete a large amount of cysteine proteases (40). The cysteine protease from S. *mansoni* can hydrolyze collagen, hemoglobin and immunoglobulin G (IgG) in vitro, and may be concerned with digestion of host tissue in pathogenesis (41, 42). Our results also showed the cysteine proteases were successfully identified in the ES proteins of plerocercoids and the proteases might come from the excretory and secretory products and the cuticles (membrane proteins), and are directly exposed to the host's immune system and are the main target antigens which induce the immune responses. Hence, the cysteine proteases from *S. mansoni* ES proteins recognized by early infection sera might be the early diagnostic antigens for sparganosis, which is needed to be further confirmed in the experiments.

The results of searches with MALDI-TOF peptide masses indicate that the proteins of other 4 spots were not included in the databases. This might mean that these proteins have not yet been described. So far, there is very limited information on genome sequence or expressed sequence tags of *S. mansoni* and only few genes coding for cysteine protease, superoxide dismutase, glyceraldehyde-3-phosphate dyhydrogenase, and α-tubulin were submitted into the GenBank database (24). The possibility of proteolysis causing these spots of relatively less protein content should also be taken into consideration. Determination of the peptide sequences in the future will be required to definitively identify the sparganum ES proteins recognized by the early infection sera.

## Conclusion

The ES sparganum proteins were analyzed by 2-DE and Western blot probed with early sera from the sparganum-infected mice at 14 days post-infection. Three of the seven immunoreactive protein spots with 23-31 kDa were successfully identified and characterized as the same *S. mansoni* protein (cysteine protease). The cysteine protease from *S. mansoni* ES proteins recognized by early infection sera might be the early diagnostic antigens for sparganosis.
